# Enhanced protein secretion in reduced genome strains of *Streptomyces lividans*

**DOI:** 10.1186/s12934-023-02269-x

**Published:** 2024-01-05

**Authors:** Mohamed Belal Hamed, Tobias Busche, Kenneth Simoens, Sebastien Carpentier, Jan Kormanec, Lieve Van Mellaert, Jozef Anné, Joern Kalinowski, Kristel Bernaerts, Spyridoula Karamanou, Anastassios Economou

**Affiliations:** 1 Department of Microbiology, Immunology and Transplantation, Rega Institute, Laboratory of Molecular Bacteriology, KU Leuven, Herestraat 49, Leuven, B-3000 Belgium; 2 Molecular Biology Depart, National Research Centre, Dokii, Cairo, Egypt; 3 Center for Biotechnology (CeBiTec), Bielefeld University, Bielefeld, Germany; 4 Department of Chemical Engineering, Chemical and Biochemical Reactor Engineering and Safety (CREaS), KU Leuven, Leuven, B-3001 Belgium; 5 SYBIOMA, KU Leuven facility for Systems Biology Based Mass Spectrometry, Leuven, B-3000 Belgium; 6 Institute of Molecular Biology, Slovak Academy of Sciences, Dubravska cesta 21, Bratislava, 84551 Slovakia; 7 Department of Neurosciences, Leuven Research Institute for Neuroscience and Disease (LIND), KU Leuven, VIB-KU Leuven Center for Brain & Disease Research, Leuven, Belgium

**Keywords:** Reduced genome strains, Secretome, Proteomics, Transcriptomics, Heterologous secretion

## Abstract

**Background:**

*S. lividans* TK24 is a popular host for the production of small molecules and the secretion of heterologous protein. Within its large genome, twenty-nine non-essential clusters direct the biosynthesis of secondary metabolites. We had previously constructed ten chassis strains, carrying deletions in various combinations of specialized metabolites biosynthetic clusters, such as those of the blue actinorhodin (*act*), the calcium-dependent antibiotic (*cda*), the undecylprodigiosin (*red*), the coelimycin A (*cpk*) and the melanin (*mel)* clusters, as well as the genes *hrdD*, encoding a non-essential sigma factor, and *matAB*, a locus affecting mycelial aggregation. Genome reduction was aimed at reducing carbon flow toward specialized metabolite biosynthesis to optimize the production of secreted heterologous protein.

**Results:**

Two of these *S. lividans* TK24 derived chassis strains showed ~ 15% reduction in biomass yield, 2-fold increase of their total native secretome mass yield and enhanced abundance of several secreted proteins compared to the parental strain. RNAseq and proteomic analysis of the secretome suggested that genome reduction led to cell wall and oxidative stresses and was accompanied by the up-regulation of secretory chaperones and of *secDF*, a Sec-pathway component. Interestingly, the amount of the secreted heterologous proteins mRFP and mTNFα, by one of these strains, was 12 and 70% higher, respectively, than that secreted by the parental strain.

**Conclusion:**

The current study described a strategy to construct chassis strains with enhanced secretory abilities and proposed a model linking the deletion of specialized metabolite biosynthetic clusters to improved production of secreted heterologous proteins.

**Supplementary Information:**

The online version contains supplementary material available at 10.1186/s12934-023-02269-x.

## Background

*Streptomyces* is a ubiquitous soil microorganism, known for its ability to secrete polypeptides and specialized metabolites [1, 2]. The lack of an outer membrane and the low level of secreted proteases makes it an attractive platform for heterologous polypeptide production [3] like tumor necrosis factor-alpha [4, 5], xyloglucanase [6], cellulase A [2], phospholipase D [7], glutenase [8] and red fluorescent protein [3].

The solved chemical structures of specialized metabolites (< 30% solved; [9]) are highly diverse and include polyketides, peptides, pyrones, oligopyrroles, γ-butyrolactones, butenolides, furans, terpenoids, fatty acids, nucleosides and deoxysugars. Various pathways are involved in the biosynthesis of these specialized metabolites including polyketide [10] as well as ribosomal and non-ribosomal peptide synthases [11, 12], shikimate [13], β-lactam [14] and carbohydrate [12] whose expression varies with environmental conditions [15]. The genes involved in specialized metabolite biosynthesis are usually clustered. The number of clusters varies between species; 37 and 29 clusters were detected *in silico* in *S. avermitilis* [16] and *S. lividans* TK24/ *S. coelicolor* A3 (2) genomes, respectively [16–18]. The clusters directing the biosynthesis of polyketides (*act*, *cpk*A), peptidic (calcium-dependent ionophore, *cda*) and hybrid peptidic/ polyketide (*red*) antibiotics are the best characterized. These antibiotics are highly produced by *S. coelicolor* and poorly produced by *S. lividans* [19]. Cluster-situated transcriptional activators, regulate expression of the various pathways [20, 21]. The most frequently encountered ones are the *Streptomyces* antibiotic regulatory proteins (SARPs) [22]. For the *act*, *red*, *cda* and *cpk* clusters the respective SARPs are ActII-ORF4, RedD, CdaR, and CpKO [16]. The transcription of ActII-ORF4 and RedD in *S. coelicolor* A3(2) is initiated by an RNA polymerase holoenzyme containing the non-essential sigma factor *hrdD* [23–25]. The upregulation of the SARPs expression coincides with morphological and physiological differentiation [26]. In *S. coelicolor*, the transcription of *redD* and *cdaR* doubles during the exponential phase, while that of *act*II-*ORF4* gene increases before entering the stationary phase [27].

Several reduced genome strains were generated to improve bacterial metabolic efficiency [28, 29] and these manipulations led unexpectedly to growth rate reduction as well as enhanced protein secretion [30]. For instance, the deletion of non-essential *E. coli* genes, in a step-wise manner (from 48 to 982 kb), revealed a positive correlation between genome shortening and slow growth [31]. In *Bacillus subtilis*, the deletion of 11 non-essential clusters (865 genes) was correlated with the enhanced secretion of heterologous cellulase and M-protease [32] and a 20.7% reduction of the genome of this strain led to two-fold increase in the secretion of alkaline cellulase Egl237 [33]. A reduction of only 2.8% of the *Lactococcus lactis* NZ9000 genome (including prophages, transposons, and related proteins) led to a two-fold increase in the secretion of red fluorescent protein [34].

Many attempts were made to construct reduced genome *Streptomyces* strains with the aim to improve precursors supply for the optimal biosynthesis of target heterologous proteins and antibiotics [35–37]. In doing so, specific proteases and specialized metabolite genes were deleted [24, 36–38]. A *Streptomyces* strain missing the zinc metalloprotease FtsH secreted 29 folds more mRFP than the original strain [37]. A *S. coelicolor* derivative strain missing 10 polyketide and non-ribosomal peptide clusters showed 6 folds enhanced production of actinorhodin compared to the original strain [39]. Another *S. coelicolor* derived strain deleted for the *act*, *red*, *cpk* and *cda* clusters showed an enhanced heterologous production of chloramphenicol and congocidin [40] or of the antitumoral polyketide mithramycin A [38].

**Fig. 1:**
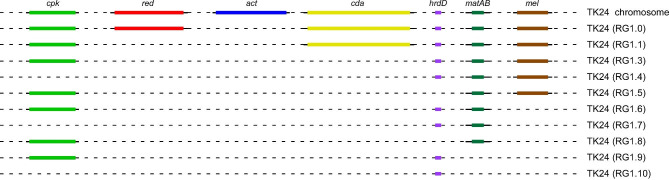
Physical maps of the chromosomal regions deleted in *S. lividans* TK24 The regions corresponding to the actinorhodin (26.7 kb), undecylprodigiosine (39.3 kb), calcium dependent antibiotic (26.7 kb), coelimycin P1 (59.1 kb) and melanin (8.9 kb) biosynthetic clusters as well as those encoding for the transcription factor HrdD (1.3 kb) and aggregation genes *mat*AB (4.5 kb) were deleted in various combinations

We have previously deleted 5 specialized metabolite clusters and 3 individual genes from *S. lividans* TK24, in various combinations, generating 10 reduced genome strains (hereafter RG; Fig. 1; Table 1 and S1) [35, 38]. The deleted clusters were those directing the biosynthesis of the specialized metabolites *cda, cpk*, *red* and *act* as well as the gene encoding for the sigma factor *hrdD* involved in their transcription (Fig. 1). Furthermore, since melanin and extracellular glycan can impact negatively the production and industrial bioprocessing of heterologous secreted proteins [38, 41], the genes responsible for their biosynthesis (*mel* and *mat*A/B respectively) were also deleted.

**Table 1:** Reduced genome strains with the indicated gene/cluster deletions. Details in Table S1

No.	Strain name	Deleted clusters and genes						
1	TK24(RG1.0)			*act*				
2	TK24(RG1.1)		*red*	*act*				
3	TK24(RG1.3)		*red*	*act*	*cda*			
4	TK24(RG1.4)	*cpk*	*red*	*act*	*cda*			
5	TK24(RG1.5)		*red*	*act*			*hrdD*	
6	TK24(RG1.6)		*red*	*act*	*cda*	*mel*		
7	TK24(RG1.7)	*cpk*	*red*	*act*	*cda*	*mel*		
8	TK24(RG1.8)		*red*	*act*	*cda*	*mel*	*hrdD*	
9	TK24(RG1.9)		*red*	*act*	*cda*	*mel*		*matAB*
10	TK24(RG1.10)	*cpk*	*red*	*act*	*cda*	*mel*		*matAB*

We show here that the secretome of these reduced genome strains differ from that of the parental strain. These strains exhibit an improved capacity to produce and secrete both native and heterologous proteins compared to the parental strain. Our study led to a better understanding of the connection between the deletion of specialized metabolite clusters and the enhanced production of secreted proteins.

## Materials and methods

### Strains, media and vectors used in the study

*Streptomyces lividans* TK24 was used as a wild-type strain [2, 42]. Protoplast formation and subsequent transformation were as described [43, 44]. Deletion of specialized metabolite clusters to generate reduced genome strains (RG) was carried out as described [35, 38].

Phage medium, Minimal Medium (MM), Minimal Medium with 5 g Bacto casamino acids/L (MM_C5_) and Nutrient Broth (NB) without NaCl, were as described [2, 3, 45]. Solid medium MRYE was as previously described [44]. Whenever necessary, media were supplemented with thiostrepton (for liquid 10 µg/µL ; for solid 50 µg/ µL) [2] .

### Growth conditions

*S. lividans* TK24 and derivatives were incubated in Phage medium (50 ml) supplemented with thiostrepton (10 µg/ml), at 28 °C, for 48 h, with continuous shaking (240 rpm). The optical density (OD_600_) of precultures was measured. Mycelia were harvested (3800 x g; 15 min; SIGMA 3-16 K centrifuge), washed twice with sterile water and homogenized in 50 mL sterile water. Strains were then inoculated into 250-mL Erlenmeyer flasks containing 100 mL of nutrient broth medium (NB). To secure the same amount of mycelia per inoculum was used we followed this formula: [volume of inoculum= (Final volume of culture X 0.25)/ OD_600_] [3]. The flasks were shaken at 240 rpm, at 28 °C; the pH was controlled using 100 mM MES buffer (pH 6.9).

mRFP and mTNFα production in all strains was achieved as described in [2, 3, 6].

### Dry cell mass determination

For dry cell weight (DCW) determination, 10 mL of cultures were centrifuged (3800 x g; 15 min; SIGMA 3-16KL). Bacterial pellets were resuspended in sterile water and filtered under vacuum using pre-dried and pre-weighted 0.2 μm filters (PORAFIL® MV; Macherey-Nagel). Filters were then re-dried (12–24 h; at 60°C) and re-weighted.

### Fluorescence assays and quantification of fluorescent proteins

To evaluate strain performance, we compared the mRFP fluorescence intensity of the different cultures at their transition point to stationary phase (excitation: 550 nm; emission: 580 nm; Infinite® M200 microplate reader; Tecan). Error propagation was applied to calculate DCW-specific mRFP production.

Quantification of mRFP using purified His-mRFP was as described [3]. The amounts of mRFP detected via fluorescence assays were then compared to those quantified by western blots using antibodies against mRFP (see western blot analysis below).

### SDS-PAGE and Western blot analysis

Following centrifugation of cultures (10 min, 4200 x g, 4 °C), proteins in the spent medium were precipitated by trichloroacetic acid (TCA; final concentration of 20% w/v; 4 ^o^C). Extracellular proteins were separated by SDS-PAGE using the Precision Plus Protein™ Standard (All Blue) marker from Bio-Rad [46, 47] and visualized by silver staining or immuno-detection. mRFP and mTNFα polyclonal antibodies were raised in rabbits against lab-purified proteins at Davids Biotechnologie, Germany. Immunodetection was carried out using the GE Healthcare Amersham ECL reagents and ImageQuant LAS 4000 Imager. High-resolution images were processed using ImageJ as described in [2].

### Secretomics sample preparation and measurement

Cell removal and protein precipitation were as previously described [3, 45]. The protein pellet was solubilized in 8 M Urea − 1 M ammonium bicarbonate solution (ABS). Protein concentration was measured using the Bradford reagent. Polypeptides (3 µg) were separated by 12% SDS-PAGE and visualized by silver staining (Shevchenko et al. 1996).

### Analysis of secretomes by nanoLC-MS/MS

A volume corresponding to the secreted polypeptides derived from 3 × 10^6^ cells (usually a volume equivalent to 20–40 µL of the initial cell culture) was used for in-solution tryptic digestion. The protein solution was initially diluted into urea (2 M final concentration in 50 mM Ammonium bicarbonate solution (ABS), followed by reduction of cysteines with 1 mM DTT (45 min; 56 ^o^C), alkylation using 10 mM Iodoacetamide (IAA) (45 min; 22 ^o^C; dark) and digestion using 0.015 µg Trypsin for 1.5 µg protein (Trypsin Gold, Promega, Fitchburg, Wisconsin; ratio trypsin/protein 1/100; overnight; 37 ^o^C). Digested peptide solutions were acidified with trifluoroacetic acid (TFA) to pH < 2, desalted using STAGE tips [48, 49], and stored lyophilized at -20 ^o^C, until the MS analysis.

Lyophilized peptide samples were re-suspended in an aqueous solution containing 0.1% v/v formic acid (FA) and 5% v/v Acetonitrile (ACN) and analyzed using nano-Reverse Phase LC coupled to a QExactive Hybrid Quadrupole - Orbitrap mass spectrometer (Thermo Scientific, Bremen, Germany) through a nanoelectrospray ion source (Thermo Scientific, Bremen, Germany). Peptides were initially separated using a Dionex UltiMate 3000 UHPLC system on an EasySpray C18 column (Thermo Scientific, OD 360 μm, ID 50 μm, 15 cm length, C18 resin, 2 μm bead size) at a flow rate of 300 nL min^− 1^. The LC mobile phase consisted of two different buffer solutions, an aqueous solution containing 0.1% v/v FA (Buffer A) and an aqueous solution containing 0.08% v/v FA and 80% v/v ACN (Buffer B). A 60 min multi-step gradient was used from Buffer A to Buffer B as follows [0–3 min constant (96:4), 3–15 min (90:10); 15–35 min (65:35); 35–40 min (35:65); 40–41 min (5:95); 41–50 min (5:95); 50–51 min (95:5); 51–60 min (95:5)].

The separated peptides were analyzed in the Orbitrap QE operated in positive ion mode (nanospray voltage 1.5 kV, source temperature 250 °C). The instrument was operated in data-dependent acquisition (DDA) mode with a survey MS scan at a resolution of 70,000 FWHM for the mass range of m/z 400–1600 for precursor ions, followed by MS/MS scans of the top 10 most intense peaks with + 2, +3 and + 4 charged ions above a threshold ion count of 16,000 at 35,000 resolution. MS/MS was performed using normalized collision energy of 25% with an isolation window of 3.0 m/z, an apex trigger 5–15 s and a dynamic exclusion of 10 s. Data were acquired with Xcalibur 2.2 software (Thermo Scientific).

Raw MS files were analyzed by the MaxQuant v1.5.3.3 proteomics software package (Cox, Mann 2008). MS/MS spectra were searched by the Andromeda search engine against the Uniprot *S. lividans* TK24 proteome (taxonomy: 457,428, last modified 2020, 7520 protein entries; [17] and common contaminants (e.g. trypsin, keratins). Enzyme specificity was set to trypsin and a maximum of two missed cleavages were allowed. Dynamic (methionine oxidation and N-terminal acetylation) and fixed (S-carbamidomethylation of cysteinyl residues) modifications were selected. Precursor and MS/MS mass tolerance was set to 20 ppm for the first search (for the identification of the maximum number of peptides for mass and retention time calibration) and 4.5 ppm for the main search (for the refinement of the identifications). Protein and peptide false discovery rate (FDR) were set to 1%. FDR was calculated based on the number of spectra matched to peptides of a random proteome database (reversed sequence database) in relation to the number of spectra matching to the reference proteome. Peptide features were aligned between different runs and masses were matched (“match between runs” feature), with a match time window of 3 min and a mass alignment window of 20 min. Protein quantification was performed using the iBAQ algorithm [50] through MaxQuant software. Differentially abundant proteins were selected using the t-test and by comparing the fold difference of average protein intensities between the samples. P-values were further corrected for multiple hypothesis testing error using the Benjamini-Hochberg method [51].Thresholds for the analysis were set to adjusted p-value < 0.05 and fold difference > 2. Functional characterization of proteomics results was performed after filtering the dataset only to secreted proteins, excluding cytoplasmic contamination, using proteome annotation as described in the SToPSdb [52] (www.stopsdb.eu). The percentage of differentially abundant proteins that match a specific term over the total differentially abundant proteins for each condition was plotted. Keywords were derived after manual curation of the proteome.

### RNA isolation and differentially expressed genes

Samples for transcriptomics data analysis were taken during the late-exponential growth phase. The cells were grown in NB medium and the harvesting and RNA isolation was performed using Trizol reagent (Invitrogen) following the manufacturer’s instructions, and was treated with DNase I (Invitrogen) to remove chromosomal DNA contamination [53]. Samples of 5 different biological replicates for each strain were isolated separately and pooled after quality control. The RNA quality was checked, and the TruSeq Stranded mRNA Library Prep Kit was done as described in [45, 54, 55].

Transcriptomics analysis and differential expression were carried out as described in [56]. In brief raw FASTQ files were processed using the CLC Genomics Workbench (CLC Bio, Aarhus, Denmark). Raw reads were trimmed by their overall quality (score: 0.05; maximum ambiguous nucleotides: (2) and length (minimum length: 15 nucleotides). The filtered reads were mapped to *S. lividans* TK24 genome sequence, accession number (NZ_CP009124), with the default parameters (mismatch cost: 2; insertion cost: 2; deletion cost: 3; length fraction: 0.9; similarity fraction: 0.9; and ignore non-specific matches). The read counts were normalized using the DESeq-2 package in R [57].

Reads per kilobase per million mapped reads (RPKM) [58] were calculated based on the raw read counts per CDS. Differential gene expression analysis was performed using ReadXplorer v2.2 [59] using DESeq2 [57]. The signal intensity value (A-value) was calculated by the average (log_2_) RPKM of each gene and the signal intensity ratio (M-value) by the difference of (log_2_) TPM. The differential RNA-Seq data was evaluated using an adjusted P-value cut-off of P ≤ 0.05 and a signal intensity ratio (M-value) cut-off of ≥ + 1 or ≤ − 1 (fold-change of ± 2).

### Metabolomics analysis

Exometabolomics analysis was performed on triplicate cultures of *S. lividans* TK24 wildtype and RS1.9 grown in nutrient broth. At several time points, free amino acid concentrations in the medium were measured. Cells were removed by centrifugation (4500 x g for 5 min) followed by microfiltration (syringe filter, 0.2 μm, cellulose acetate). Free amino acids were determined with the EZ:FAAST amino acid analysis kit of Phenomenex on a GC-FID (Perkin Elmer) according to supplier instructions. Measured amino acids are: alanine (ALA), glycine (GLY), α-amino-butyrate (ABA), valine (VAL), β-amino-isobutyric acid (BAIB), leucine (LEU), isoleucine (ILE), threonine (THRE), serine (SER), proline (PRO), asparagine (ASN), aspartate (ASP), methionine (MET), hydroxyproline (HYP), glutamate (GLU), phenylalanine (PHE), α-amino-adipic acid (AAA), glutamine (GLN), ornithine (ORN), cysteine (CYS), lysine (LYS), histidine (HIS), tyrosine (TYR), tryptophan (TRP). BAIB, HYP, AAA, GLN, ORN, and CYS were left out of the PCA analysis as these data were either too noisy or the amino acids were not really used as substrates. Principal component analysis was performed in MATLAB R2016b (The Mathworks) using included functions.

### Miscellaneous

Chemicals (Sigma), Bacto Soytone (DIFCO Laboratories), DNA enzymes (New England Biolabs) and oligonucleotides (Eurogentec) were used.

The mass spectrometry proteomics data have been deposited to the ProteomeXchange Consortium via the PRIDE partner repository [60] with the dataset identifiers PXD040146.

Submission details:

Project Name: Enhanced protein secretion in reduced genome strains of Streptomyces lividans.

Project accession: PXD040146.

Reviewer account details:

Username: reviewer_pxd040146@ebi.ac.uk.

Password: 1uBKUTp6.

## Results

### Transcription of specialized metabolite clusters/genes in *S. lividans* TK24

The transcription levels of the clusters/ genes that had been deleted in the RG strains (Fig. 1) were monitored in the *S. lividans* TK24 parental strain during growth in minimal medium supplemented with glucose [2, 3, 24, 37]. Samples were collected at three distinct growth phases (early-/ late-exponential and stationary) and transcriptomes analysed using RNAseq. Results are presented in Fig. S1 and Table S2. Under these conditions, the transcript level for most of the specialized metabolite biosynthetic genes was high at the late-logarithmic phase (Fig. S1). The *act* cluster (*SLIV_12925* to *SLIV_13030*) showed the highest transcript levels in both late-exponential and stationary phases. Specialized metabolite regulatory proteins (SARPs), antibiotics cluster activators [61], *red*Z (*SLIV_09200*), *red*D (*SLIV_09220*) and *cda*R (*SLIV_21605*) showed high transcript levels in the late-log phase (in red). The *act*II-*ORF4* (*SLIV_12960*) showed the highest transcript levels in both late-exponential and stationary phases (in red). On the other hand, *cpk*O (*SLIV_06745*) and *cpk*N (*SLIV_06705*), showed moderate transcript levels during all growth phases (in red). The upregulation of SARPs is consistent with previous reports in *S. coelicolor* A3(2) [62], despite differences in the level of expression among the two strains [63].

### Effect of cluster/gene deletions on cell growth and native secretome

**Fig. 2:**
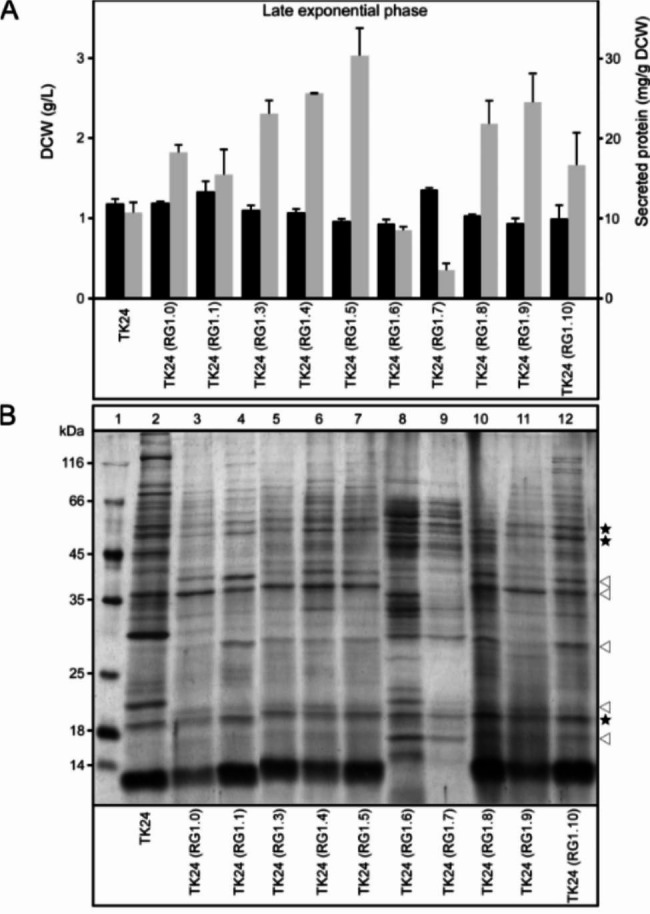
Total biomass and native secretome yield in *S. lividans* TK24 and reduced genome derivative strains. **(A)** Dry cell weight (DCW; g/L; black bars) and total secretome yield (mg secreted protein/ g DCW; grey bars) following growth of the indicated strains in nutrient broth (NB) until late exponential phase. *n* = 3; values represent the mean ± SD; **(B)** Equal amount of secreted polypeptides (3 µg/lane) from the indicated strains, grown as in A, were analyzed by SDS-PAGE and silver stained. Lane 1: molecular weight marker. Proteins with major (arrows)/ little (asterisks) change among strains are indicated

The effect of cluster/gene deletions on cell growth was tested for all the engineered strains. We used nutrient broth (NB) as it has been previously described as the best-performing medium for native and heterologous protein production [2, 3, 37]. For each strain, the same amount of mycelia was used as inoculum (see formula in Materials and methods). Most strains showed a growth pattern similar to that of the parental strain (Fig. S2A). However, 7 among them yielded slightly lower and 2 yielded slightly higher amount of dry cell weight (DCW; g/L), at late exponential phase, than the parental strain (Fig. 2A). The slower growth rate of some RG strains might be attributed to the rapid consumption/ eventual depletion from the medium, of aspartate, glutamate and asparagine, as seen for RG1.9 (Fig. S5; Table S7).

To determine the effect of cluster/gene deletions on the native secretome, the amount of total native secretome produced by each strain per gram of dry cell weight (DCW) was estimated. The secretome mass produced by 8 of the 10 strains was higher than that produced by the parental strain (Fig. 2A). The highest mass was obtained from RG1.5 and was 3 folds higher than that of the parental strain (~ 30 mg/g DCW). RG strains 1.3, 1.4, 1.8 and 1.9 produced 2 folds more, whereas RG strains 1.6 and 1.7 produced less, secretome mass than the parental strain (Fig. 2A). Our results confirm previously reported correlation between slow growth rate and enhanced protein secretion [2, 3, 45].

To study the impact of deletions on the native secretome profile, following growth in NB medium for 24 h, the secreted polypeptides of all 11 strains were TCA precipitated, analyzed by SDS-PAGE and silver stained (Fig. 2B). While the abundance of some proteins was similar between strains (asterisks), that of others changed in some RG strains (arrows). Some new polypeptides appeared in RG strains while others were lost.

**Fig. 3:**
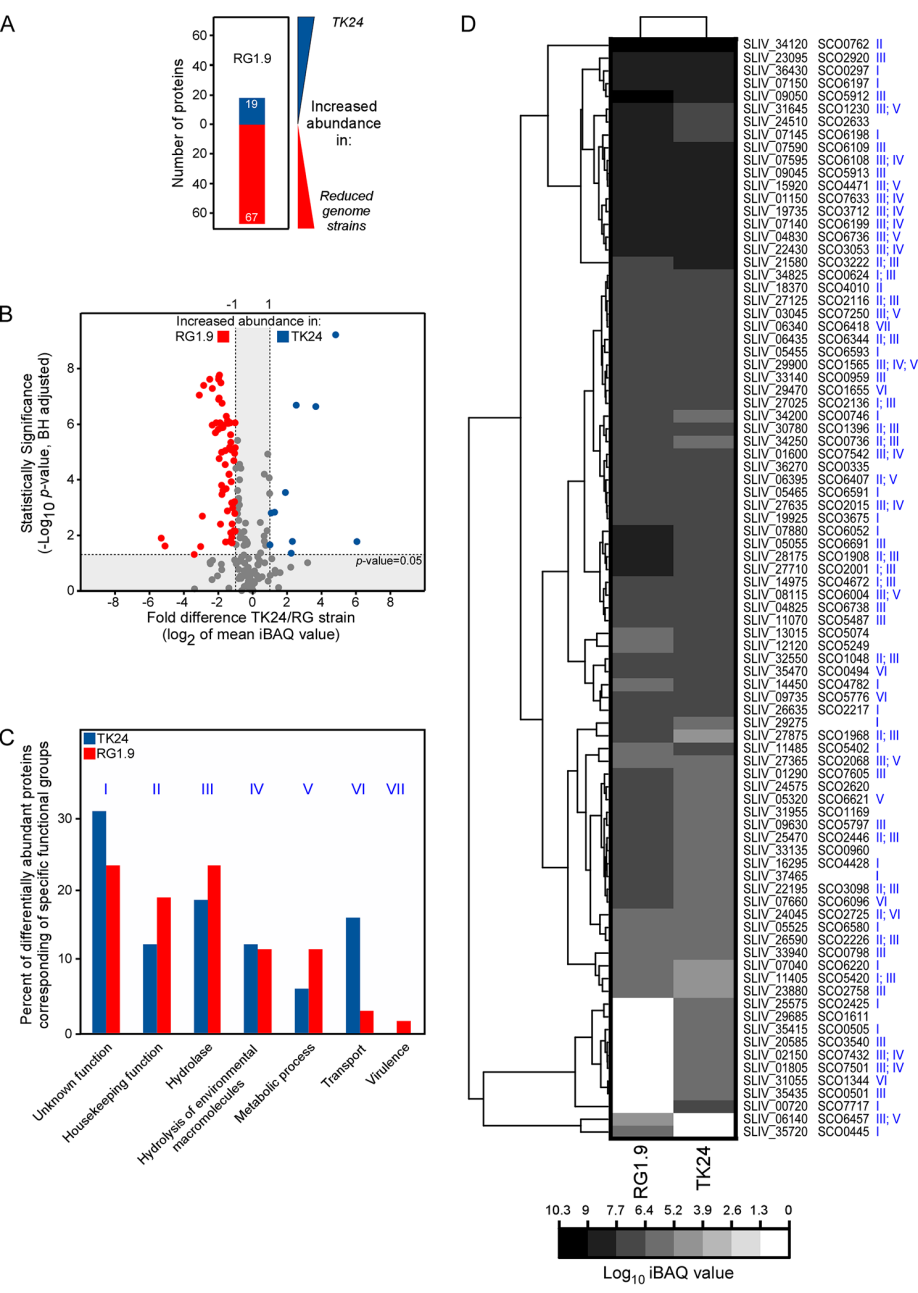
Comparative secretome analysis of *S. lividans* TK24 and RG1.9 strains. **(A)** Proteins secreted by the parental and by the RG1.9 strains were differentially abundant. Blue: more abundant in the parental strain; red: more abundant in the RG1.9 strain. The secretome amount that was produced by a fixed amount of cell biomass was loaded for both strains for proteomics analysis. **(B)** The summary of proteins that were differentially abundant in the secretome of the parental and the RG1.9 strains is shown as volcano plots (for detailed description see panel C and Table S1). Each dot represents one protein. Blue: more abundant in the parental strain; Red: more abundant in the RG1.9 strain. Plotted on the *x* axis is the fold difference (in log_2_ scale) of the mean protein abundance in the parental strain over that in RG1.9, and on the *y* axis the *p*-value derived from a *t*-test between the two strains (–log_10_, adjusted from [64]. **(C)** Classification of the differentially abundant proteins based on their biological function, as described [52]. The ratio ‘number of differentially abundant proteins that belong to the indicated functional group/ total number of differentially abundant proteins in the same strain’ is plotted. The dataset was filtered in order to retain secreted proteins and eliminate contaminating cytoplasmic proteins. Functional groups are coded with latin numbers as indicated. **(D)** Clustering of iBAQ values for proteins that showed differential abundance in the secretome of the parental and of the RG1.9 strains. Proteins have been annotated with both *S. lividans* TK24 (SLIV) and *S. coelicolor* (SCO) gene IDs. Latin numbers next to gene IDs indicate protein functions as these were grouped in panel C

To accurately determine the nature of the changes, the secretomes of the parental strain (TK24) and of three RG strains (1.4, 1.5, 1.9; that produced the highest native secretome mass; Fig. 2A) were compared by label-free nanoLC-MS/MS. Polypeptides were identified and quantified (Fig. 3 and S3; Tables S3 and S4). The abundance of each protein (units/ mg DCW) in the RG strains was compared to that of the parental strain (Fig. 3 and S3). Several secreted polypeptides (~ 10–15% of a secretome) were statistically differentially abundant (lower or higher); i.e. 62 proteins in RG1.4, 55 in RG1.5 and 86 in RG1.9. Out of them, 39 proteins in RG1.4, 36 in RG1.5 and 67 in RG1.9 were more abundantly secreted in the RG strains than in the parental strain (Fig. 3 and S3; Table S4). Examples of proteins whose secretion levels were affected include a putative lipoprotein of the LolA/LolB/LppX family and a N-acetylmuramoyl-L-alanine amidase that were secreted 1.6 and 3 folds more, respectively, by all of the RG strains than by the parental one. Phospholipase A2 (D6ES51) and A0A076MBH5 of unknown function secreted through the Tat pathway, were secreted respectively 1.5 and 6 folds less by the RG strains than by the parental strain.

The secreted proteins that showed differential abundance between mutants and parental strain fell into six main functional classes (Fig. 3C and D and S4). Proteins with hydrolytic functions were oversecreted in all RG strains examined [e.g. Amidase (*SLIV_06435*), putative phosphodiesterase (IPR017946) (*SLIV_27875*) and probable subtilinase-type protease inhibitor (*SLIV_34120*)] (RG1.4, 1.5, 1.9; Fig. 3C and D and S4). Hydrolases, peptidases and proteases were mainly over-secreted in RG1.9. These include two metallopeptidases (*SLIV_01290* and *SLIV_04830*), two proteases (SLIV_09050 and *SLIV_09045*), a peptidase (*SLIV_04650*) containing LysM (IPR018392) and M23 (IPR016047) domains, two glycoside hydrolases (*SLIV_33135* and *SLIV_34355*) (Fig. 3C and S4; Table S4). All subsequent experiments were done with the RG1.9 strain that exhibited the highest number of differentially abundant proteins compared to the parental strain.

Our proteomics analysis revealed that the RG1.9 strain over-secreted 40 proteins with hydrolytic function including 13 proteins that are likely to target the cell wall. More specifically these include: a D-Ala-D-Ala dipeptidase (D6EQU5), a protein with lysozyme-like (IPR023346) (D6EKB6) and LysM (IPR018392) domains (D6ETM1) and a gamma-glutamyltransferase (D6EID2) that were secreted *via* the Sec pathway, and the beta-N-acetylhexosaminidase (A0A076M9V2) and L, D-transpeptidase (D6EYE3) that were secreted *via* the Tat pathway (Tables S3 and S4). The RG1.9 strain also over-secreted a protein with redox-related function. Collectively, our observations suggest that the *S. lividans* derived strains bearing deletions of the genes mentioned above have an enhanced ability to secrete hydrolytic proteins involved in the remodeling of the cell wall.

### Effect of cluster/gene deletions on transcription

The differences between the native secretome profiles of the parental *S. lividans* TK24 and the RG strains could be explained by enhanced transcription of genes encoding the secreted proteins and/or of genes encoding chaperones or components of the secretion apparatus.

**Fig. 4:**
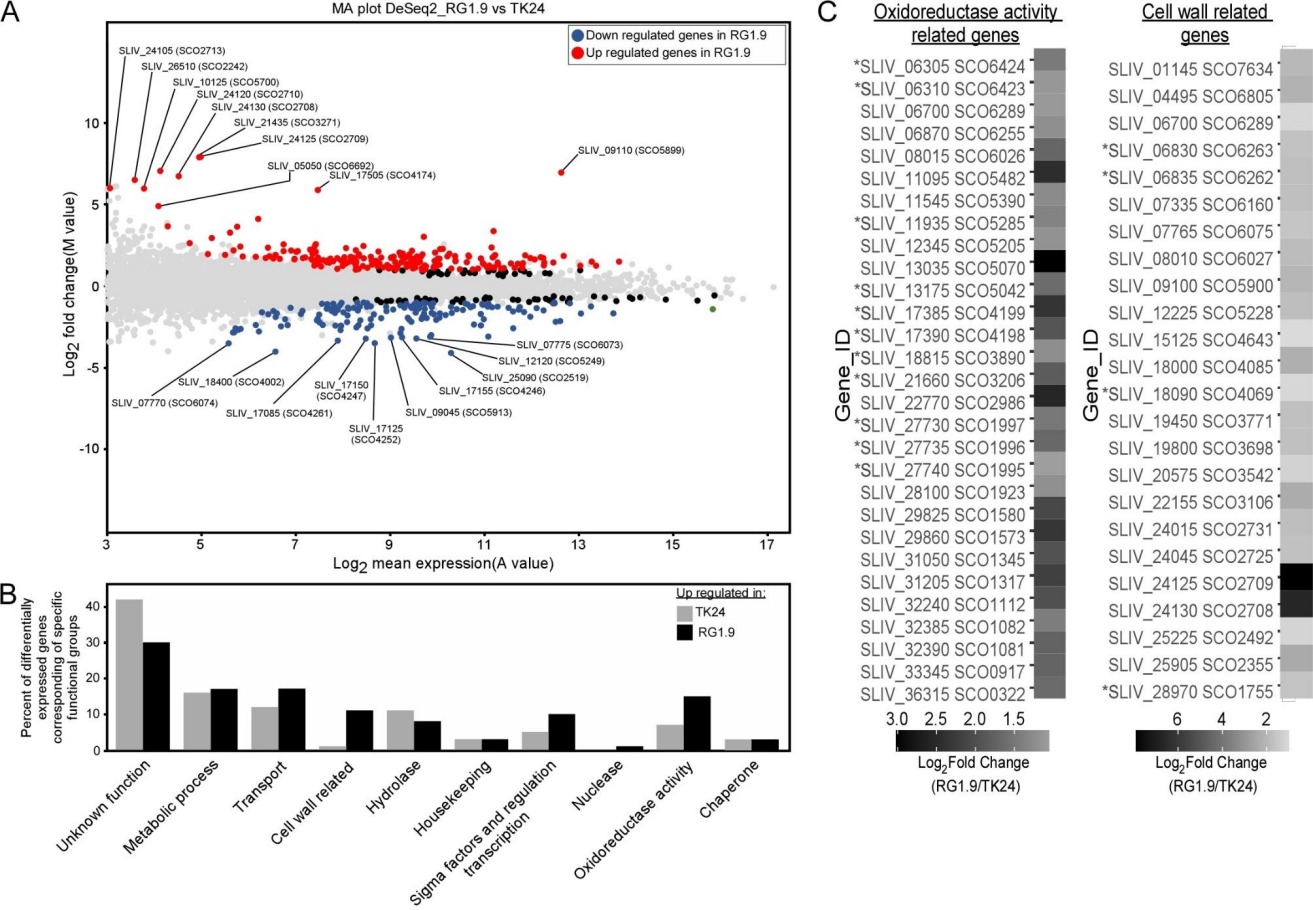
Differential expression of genes between *S. lividans* TK24 and RG1.9 strains. **(A)** MA-plot for RNA-Seq datasets comparing *S. lividans* TK24 and RG1.9 strains. Genes similarly regulated in both strains (grey) and up- (red) or down- (blue) regulated in RG1.9 are indicated (M > 1 or M <-1 respectively; adjusted *p*-value < 0.05). M and A values were calculated as indicated (Material and Methods). For the 10 most prominently up- / down- regulated genes their SLIV/SCO gene IDs are indicated. **(B)** Differential expression of genes encoding proteins with the indicated biological function (as described, [52]) in *S. lividans* TK24 (grey) and RG1.9 (black) strains. For each strain and each functional group, the ratio ‘number of differentially expressed genes / total number of genes’ was plotted. **(C)** Heatmap of genes related to oxidoreductase (left) or cell wall (right) functions that were upregulated in RG1.9, classified as in panel B. Annotations with SLIV/ SCO gene IDs are indicated. Asterisks: genes that belong to the Sig^R^ or Sig^E^ regulons

In order to clarify these points, we compared the transcriptomes of the parental and RG1.9 strains, both grown in NB. 349 genes out of a total of 7306 genes showed differential expression between the two strains (signal intensity ratio M-value > 1; adjusted *p*-value < 0.05; see materials and methods; Fig. 4; Table S6). Out of these, 196 were upregulated in RG1.9, encoding 132 cytoplasmic, 40 membrane, 5 nucleoid/ ribosomal and 14 secreted proteins. All genes that showed differential expression between the parental and the RG strain were classified into 10 functional groups; 6 of them were upregulated in RG1.9 (Fig. 4B, Table S6).

Interestingly, a gene encoding for the bifunctional translocase subunit SecDF, an important component of the Sec export system, was upregulated 1.8-folds in RG1.9 (Table S6) whereas none of the Tat-pathway component genes were affected. Furthermore, four genes encoding proteins involved in stress response.

were significantly upregulated (up to 1.5 fold) in RG1.9 (Table S6). These include the putative proteasome assembly chaperone 2 (IPR019151), the ATP-dependent serine protease Lon (D6EUK1) [65], the thioredoxin reductase A (D6EMA7) [66] and the nitrate reductase subunit delta (D6EHT8) belonging to the OsdR regulon [67] (Table S6).

Interestingly, among the genes upregulated in the RG strain 20 were related to cell wall function [e.g. the genes encoded for Peptidoglycan biosynthesis protein MurJ (IPR004268) (*SLIV_24125*) and O-antigen ligase-related (IPR007016) (*SLIV_24130*)] (Fig. 4B-C; Table S6; marked with one asterisk). Four of them are known to be under the control of the sigma factor Sig^E^, involved in the cell envelope stress response [68]. Eighteen additional genes encoded proteins annotated as oxidoreductase activity (Fig. 4B-C; Table S6; marked with two asterisks); 11 of them belonged to the Sig^R^ regulon involved in redox homeostasis [69]. Finally, four genes of the arginine biosynthesis pathway (Table S6; marked with three asterisks) that forms arginine from glutamate [70], showed upregulation [1.3–2.3 folds; *argH* (*SLIV_29875)*, *argC* (*SLIV_29825*), *argJ* (*SLIV_29830*) and *argG* (*SLIV_04065*)]. These genes may play a key role in cell-wall biosynthesis, since glutamine derived from glutamate is a major component of peptidoglycan. Alternatively, one cannot exclude that arg biosynthesis is required for the enhanced efficiency of the Twin-arginine translocation pathway in *S. lividans* [71–75]. These data indicated that deletion of *red*, *act*, *cda* and *mel* clusters and of the *matAB* genes in the RG1.9 strain led to cell wall and oxidative stresses.

### Effect of cluster/gene deletions on heterologous protein secretion

Finally, we tested whether genome reduction impacted secretion of heterologous polypeptides. The monomeric red fluorescence protein, tagged with a histidine tag and fused to the *vsi*-encoded signal sequence, was cloned behind the *vsi* promoter in the high copy number plasmid pIJ486 to produce pIJ486/*sp*^*SecV*^*-mRFP* [3]. This plasmid was transformed via protoplast transformation (see materials and methods) in *S. lividans* TK24 and two RG strains (1.5 and 1.9; both yielded the highest secretome mass; Fig. 3A).

**Fig. 5:**
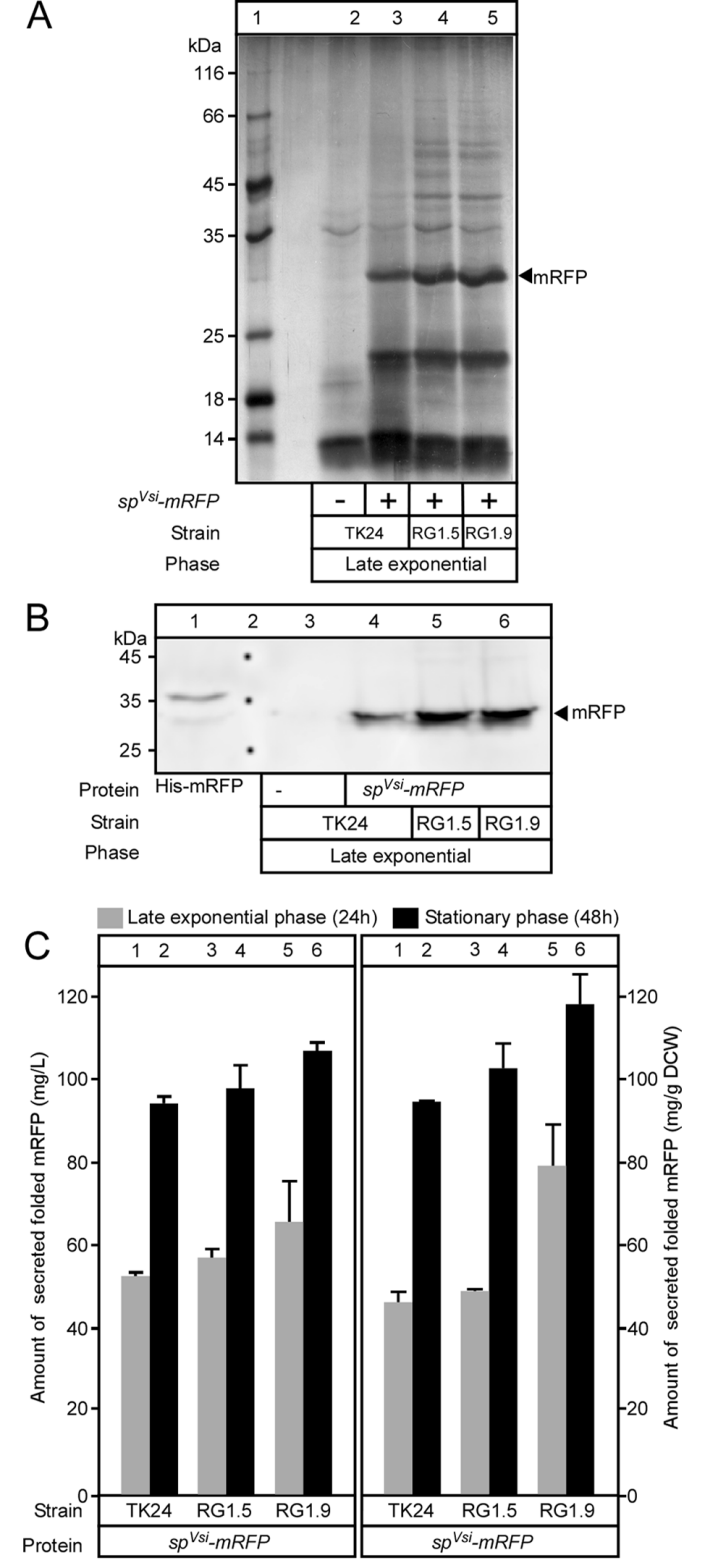
Effect of cluster/gene deletions on heterologous protein secretion. **(A)** Strains carrying pIJ486/*sp*^*Secv*^*-mRFP*, as indicated, were grown in NB for 24 h. Secreted proteins were TCA precipitated, analyzed by SDS-PAGE and silver stained. Lanes were loaded with 5–10 µL of collected polypeptides, a volume corresponding to 0.1 mg DCW. Lane 1: Molecular weight marker; Arrow; mRFP, as indicated. **(B)** Immunostaining of the samples presented in Panel A (same sample loading) using mRFP specific antibodies. Lane 1: purified his-mRFP; Lane 2: molecular weight marker. **(C)** The amounts of mRFP secreted by the *S. lividans* TK24 and RG strains (as indicated), grown in NB medium, for 24 (grey) or 48 h (black bars), were expressed either in mg/L (left panel) or in mg/gram of DCW (right panel). *n* = 3, values represent the mean ± SD

Analysis of the spent growth medium of the parental and the RG strains by SDS-PAGE, showed a prominent band of ~ 27 kDa, close to the theoretical mRFP mass (25.4 kDa) (Fig. 5A; arrow) that was recognized by a rabbit polyclonal antibody raised against purified mRFP (Fig. 5B). To quantify mRFP secretion, His-mRFP, purified by metal affinity chromatography, was used to generate standard curves. These correlated either fluorescence measurements to protein mass (used in the left part of Fig. 5C) or signal intensity from immunodetection to protein mass (used in the right part of Fig. 5C) (see materials and methods; [3]). Using either quantification methods, we found that more mRFP was secreted by the RG strains than by the parental strain. After 24 h growth in NB medium, RG1.5 (51 mg/g DCW) and RG1.9 (79 mg/g DCW) secreted 25 and 70% respectively more mRFP than the parental strain (46 mg/g DCW) (Fig. 5C, right panel, compare lanes 3 and 5 to lane 1). After 48 h of growth, mRFP was only 8.5 and 24.5% more secreted by RG1.5 and RG1.9, respectively, than by the parental strain (Fig. 5C; right panel, compare lanes 6 and 4 to lane 2).

Using the same strategy we tested the secretion of mTNFα in the same strains. *S. lividans* TK24 and RG (1.5 and 1.9) strains expressing *sp*^*SecV*^*-mTNFα*, were grown in NB medium for 24 h. Secreted proteins were TCA precipitated, analyzed by SDS-PAGE and silver-stained (Fig. S6A) or α-mTNF immunostained (Fig. S6B). To quantify the secreted protein, we generated standard curves that correlated the protein mass of purified his-mTNFα to signal intensity from immunodetection by specific antibodies [5]. The results showed that mTNFα was 11 and 31% more efficiently secreted by RG1.5 (195 mg/g DCW) and RG1.9 (230 mg/g DCW), respectively, than by the parental strain (175 mg/g DCW) (Fig. S6C).

## Discussion

Our aim was to determine whether genome reduction in *S. lividans* TK24, that involved deletions, in combinations, of non-essential specialized metabolite gene clusters, of the gene encoding the sigma factor *hrdD* [68, 76, 77] and of the locus *matAB* encoding the putative polysaccharide synthases [77, 78] enhanced the overall secretome as well as the secretion of heterologous proteins.

Among the ten genome reduced strains we examined (Table 1) seven exhibited a minor reduction of biomass yield (Fig. 2A). The latter could be attributed to the rapid consumption and eventual depletion, by mid-exponential phase, of aspartate, glutamate and asparagine (Fig. S5; Table S7). For six of these strains, reduced biomass correlated with an overall enhanced ability to secrete proteins. Qualitative and quantitative changes were seen in the secretome of the RG strains compared to the parental one (Figs. 2 and 3 and S3; Table S3 and S4). The RG strains secreted 2–3 folds more polypeptides (Fig. 2A). In two of these RG strains (1.5 and 1.9) we recorded an increase in the secretion of two heterologous proteins (mRFP and mTNFα) compared to the parental strain (Fig. 5 and S5). Our data indicated that the stimulation of the secretion of native proteins also benefited to the secretion of heterologous proteins.

To gain insight into the metabolic changes caused by the combination of the various gene deletions, we performed comparative transcriptomics analysis of the parental and of one of the RG strains (RG1.9). This comparison revealed that 24 genes encoding proteins related to cell wall remodeling (biosynthesis/degradation) were upregulated in RG1.9 (Fig. 4B; Table S6). Among the 24 upregulated genes, four belonged to the Sig^E^ regulon, that is known to control positively the expression of genes involved in cell wall synthesis [68] and negatively that of specialized metabolite biosynthetic clusters, like that of actinorhodin (Table S6). *sig*^*E*^ deletions resulted in cell wall stress and actinorhodin overproduction in *S. coelicolor* [24, 79]. Consistently, this strain oversecreted > 13 proteins involved in cell wall remodeling as well as 3 proteins involved in stress response (Fig. 3B and D; Table S4). Twenty-nine genes encoding proteins with oxidation-reduction related function were upregulated in RG1.9. Eleven of them belonged to the Sig^R^ regulon [69] such as organic hydroperoxide resistance protein *SLIV_22770* (*SCO2986*), putative thioesterase/thiol ester dehydratase-isomerase *SLIV_11095* (*SCO5482*) and thioredoxin reductase A *SLIV_18815* (SCO3890)] (Fig. 4B; Table S6). Altogether, our data suggested that both cell wall and oxidative stresses occur in RG1.9.

Even if further analysis is required to disentangle the contribution of each cluster/ gene to the observed phenotypes, we can propose that oxidative stress might be a consequence of the deletion of either the *act* cluster or/and the *mel* gene since both actinorhodin and melanin were shown to act as antioxidants [63, 80–82]. Oxidative stress resulting from their absence might lead to cell wall damage. Alternatively, cell wall stress might have been caused by the deletion of *hrdD* or of the *matAB* locus. Indeed, in *S. coelicolor*, the transcription of *hrdD* is under the control of both *sig*^*E*^ and *sig*^*R*^, that are involved in sensing and responding to cell-wall [24] and thiol-oxidative [24] stresses, respectively, whereas the deletion of *matAB* is correlated with a thinner cell wall, lacking lamellae and patches and resulted into cell wall stress [78].

**Fig. 6:**
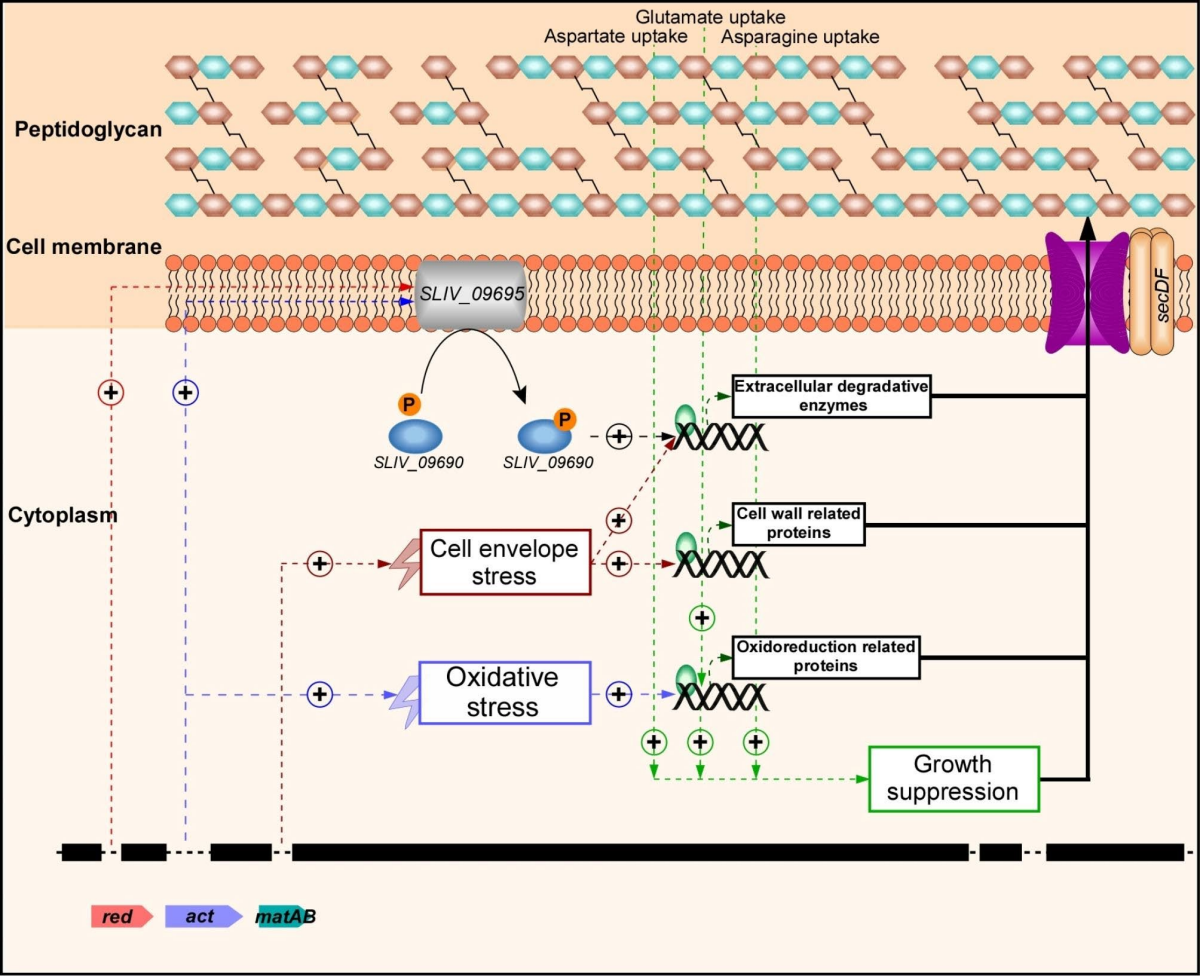
Proposed model for the effect of specialized metabolite clusters/ *matAB* deletion on protein secretion. Combined deletions of specialized metabolite clusters and *matAB* locus in *S. lividans* TK24 resulted in cells overexpressing and oversecreting proteins related to cell wall remodeling and oxidative stress. (+) indicates positive regulation

It has been proposed that Act can capture electrons of ROS/ NOS and of the respiratory chain [80]. These abilities are conferring to ACT anti-oxidant [80] and anti-respiratory functions [83]. The latter will have a negative impact on ATP generation. The deletion of ACT is thus predicted to lead to oxidative stress and enhanced ATP generation. The calcium ionophore antibiotic CDA, makes pores in the membrane and thus might dissipate the H^+^ gradient resulting in the reduction of ATP generation by the ATP synthase [62, 80]. Similarly, an ATP spilling function was attributed to undecyprodigiosin (Red) in other bacteria [80]. We propose that the deletion of the *cda*, *red* and *act* clusters would lead to oxidative stress and enhanced ATP generation that might benefit the secretion process (Fig. 6).

Furthermore, enhanced oxidative stress, resulting from the deletions of the ACT cluster, the *mel* gene and the *matAB* locus, is predicted to lead to cell wall damages that would trigger the expression of the two-component system (*SLIV_09695* and *SLIV_09690*) (Fig. 6). The latter controls positively the expression of secreted enzymes involved in the degradation of damaged cell wall components [84–86]. The necessary re-modeling of the damaged cell wall would necessitate the specific up-take and consumption of the amino acids used for peptidoglycan biosynthesis (aspartate, glutamate and asparagine). The depletion in these amino acids could explain growth rate reduction and early entry into stationary phase that was observed in RG strains (Fig. 6).

In summary, by using a combination of proteomics, metabolomics, transcriptomics and protein secretion analyses, we provided an in-depth view of the complex metabolic changes that resulted from deletions of specialized metabolite clusters and of *matAB* and *hrdD* genes. These changes are accompanied by the over expression of secretory chaperones and of components of the secretory system such as the SecDF that are likely to contribute positively to the significantly enhanced secretion of native as well as heterologous proteins. Our study revealed the existence of complex regulatory networks linking specialized metabolite production and protein secretion.

### Electronic supplementary material

Below is the link to the electronic supplementary material.

Supplementary Material 1

Supplementary Material 2

Supplementary Material 3

Supplementary Material 4

Supplementary Material 5

Supplementary Material 6

Supplementary Material 7

## Data Availability

All relevant data will be deposited in public repositories and all materials will be made available upon request.
