# Supplementary material for: Enhanced protein secretion in reduced genome strains of Streptomyces lividans
Source: Microb Cell Fact. 2024 Jan 5;23:13. doi: 10.1186/s12934-023-02269-x (PMC10768272; doi:10.1186/s12934-023-02269-x)
Supplement: Supplementary file 1 — Supplementary Material 1 [file 12934_2023_2269_MOESM1_ESM.docx]

Supplemental Information

# Enhanced protein secretion in reduced genome strains of *Streptomyces lividans*

**Hamed, M. B.^1 ,2, *^, Busche, T.^3^, Simoens, K.^4^, Carpentier, S.^5^, Kormanec, J.^6^, Van Mellaert, L.^1^, Anné, J.^1^, Kalinowski, J.^3^, Bernaerts, K.^4^, Karamanou, S.^1, 7^ and Economou, A.^1, Ϯ^**

^1^ KU Leuven, Department of Microbiology, Immunology and Transplantation, Rega Institute, Laboratory of Molecular Bacteriology, Herestraat 49, B-3000 Leuven, Belgium

^2^ Molecular Biology Depart, National Research Centre, Dokii, Cairo, Egypt

^3^ Center for Biotechnology (CeBiTec), Bielefeld University, Bielefeld, Germany

^4^ KU Leuven, Department of Chemical Engineering, Chemical and Biochemical Reactor Engineering and Safety (CREaS), Leuven B-3001, Belgium

^5^ SYBIOMA, KU Leuven facility for Systems Biology Based Mass Spectrometry, B-3000 Leuven Belgium

^6^ Institute of Molecular Biology, Slovak Academy of Sciences, Dubravska cesta 21, 84551 Bratislava, Slovakia

^7^ For correspondence: e-mail: [lily.karamanou@kuleuven.be](mailto:lily.karamanou@kuleuven.be)

^*^ Current address: Department of Neurosciences, Leuven Research Institute for Neuroscience and Disease (LIND), KU Leuven; VIB-KU Leuven Center for Brain & Disease Research, Leuven, Belgium

^Ϯ^ Deceased, 3 July 2023

Running title: Reduced genome strain secretion

Table of contents:

Supplementary figures

**Figure S1:** Transcriptome analysis of deleted genes and gene clusters

Figure S2: Reduced genome strains cell growth and native secretome production.

**Figure S3:** Comparative secretome analysis of the reduced genome strains against TK24.

Figure S4: Functional characterization of reduced genome strains secretome.

Figure S5: Exometabolomics changes between reduced genome strain *S. lividans* TK24 and RG1.9.

**Figure S6:** Effect of specialized metabolite genes clusters on heterologous proteins secretion

Supplementary tables

**Table S1:**Deleted specialized metabolite genes clusters and genes in reduced genome strains

**Table S2:**Transcriptomics results for *S. lividans* TK24 selected specialized metabolite genes clusters and genes in MM and MMCAS.

**Table S3:** Proteins identified by MS.

**Table S4:** Differentially abundant proteins.

**Table S5:** Statistical significance for identified secreted proteins TK24 and reduced genome strains.

**Table S6:** Transcriptomic analysis for *S. lividans* TK24 and RG1.9.

**Table S7:** Amino acid consumption data for TK24 and RG1.9.

Supplementary materials and methods

Transcriptomics sample preparation and analysis for TK24 in minimal medium

Supplementary Results

A reduced genome strain displays metabolic alterations

**References**


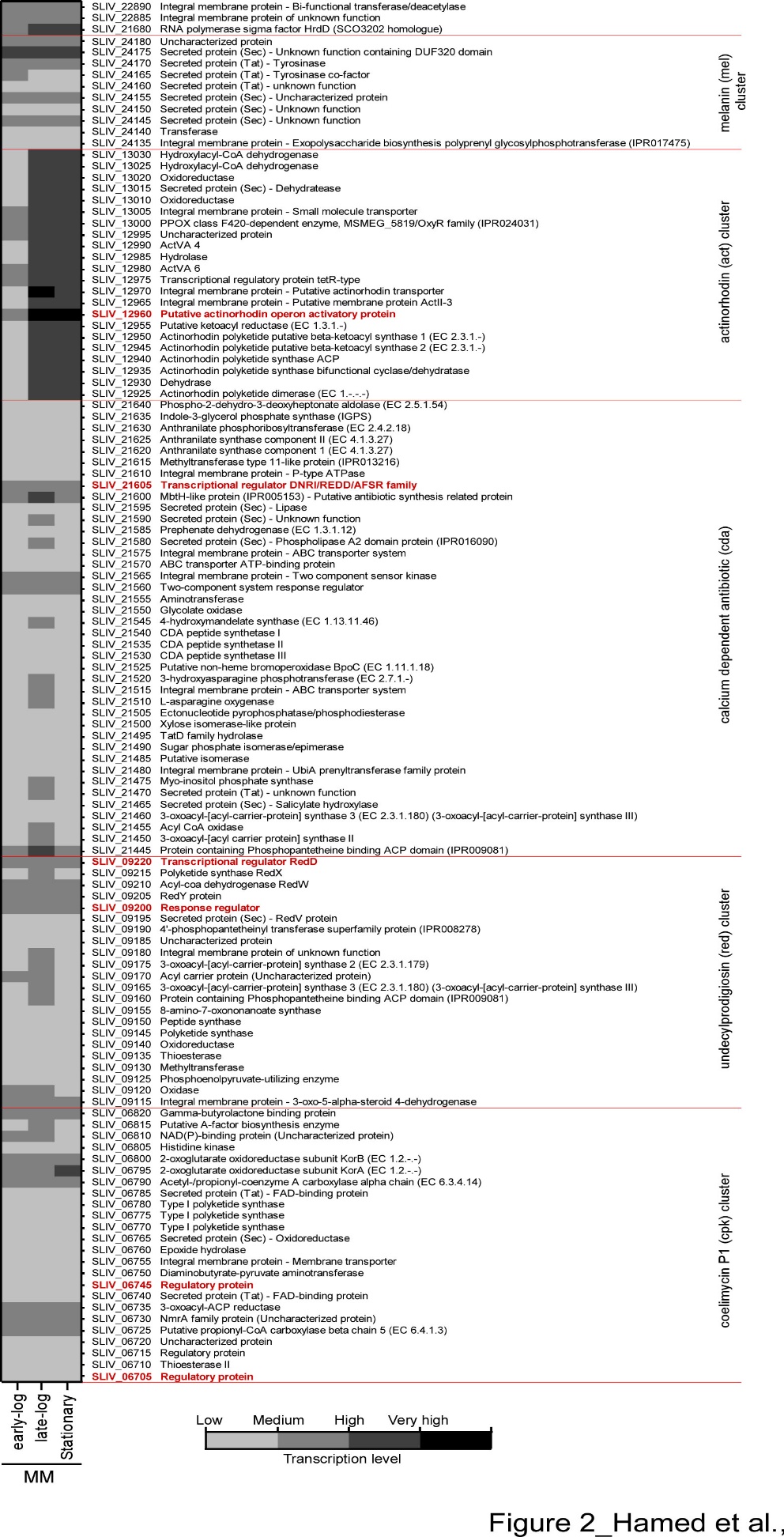
Supplementary figures

**Figure S1: Transcriptome analysis of TK24 with the emphasis on genes/clusters deleted in reduced genome strains**

Heat-map for the transcription levels of the genes/ specialized metabolites clusters that have been deleted in order to create the RG strains, in *S. lividans* TK24, grown until early/late -log and stationary phase in minimal medium (MM). Transcript abundance (TPM values; [Wagner et al., 2012](#_ENREF_7)) are log-transformed. Genes belonging to the same specialized metabolite cluster are segregated from different clusters by red lines (see Table S2). *Streptomyces* antibiotics regulatory proteins (SARPs) for each cluster are indicated by red letters. The sub-cellularly annotated proteome from SToPSdb was used ([Tsolis et al., 2018](#_ENREF_6)).


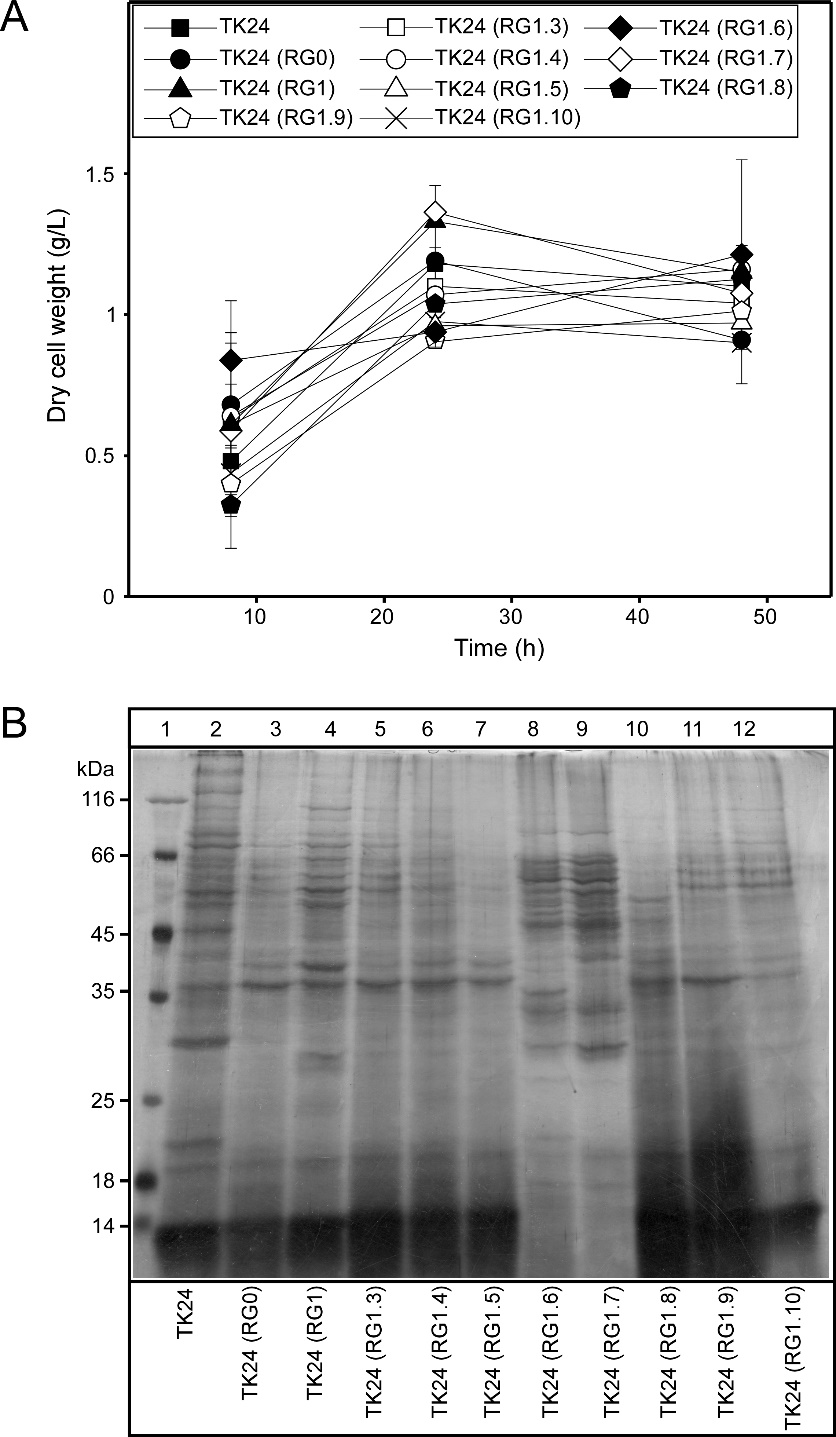


**Figure S2:** **Cell growth and native secretome production of reduced genome strains.**

**(A)** Cell growth of the reduced genome strains is compared to that of *S. lividans* TK24 in nutrient broth medium (NB) and is expressed as dry cell weight (DCW) values (g/L). *n*=3; mean ± SD.

**(B)** Polypeptides from culture supernatants (2-16 µL/lane; equivalent to 0.2 mg of cells) of the reduced genome strains or the parental strain (as indicated), were grown in nutrient broth (NB), analyzed by SDS-PAGE and silver-stained.


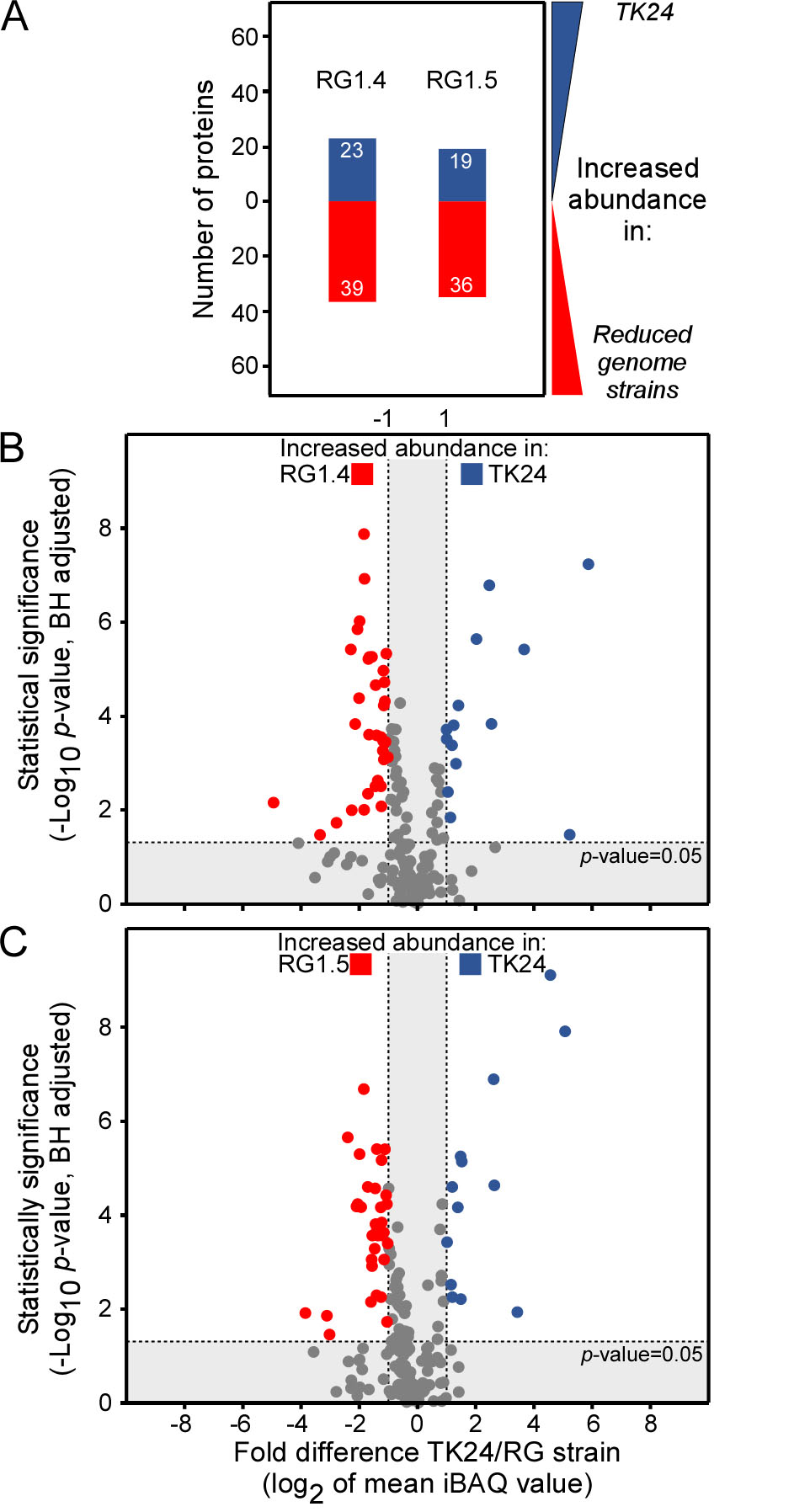


**Figure S3:** **Comparative secretome analysis between the indicated reduced genome strains and TK24 strain.**

**(A)** Number of differentially abundant secreted proteins between the reduced genome strains (1.4; 1.5) and TK24 strain. Proteins more abundant in TK24 are shown in blue; proteins more abundant in the RG strains in red. For proteomic analysis samples were normalized to the same amount of cell biomass.

**(B)** and **(C)** Volcano-plots showing the differentially abundant proteins in TK24 and reduced genome strains 1.4 **(B)** and 1.5 **(C)**. Each dot represents one protein. On the *x* axis is plotted the fold difference (in log_2_ scale) of the mean protein abundance in the TK24 (marked: TK24) over that in RG1.4 and RG1.5; on the *y* axis the *p*-value derived from a *t*-test between the two strains (–log_10_, adjusted by the Benjamini–Hochberg method). Proteins more abundant in TK24 are in blue; proteins more abundant in the reduced genome starins are in red [RG1.4 **(B)**; RG1.5 **(C)**]. Details for the differentialy abundant proteins are in Table S1.


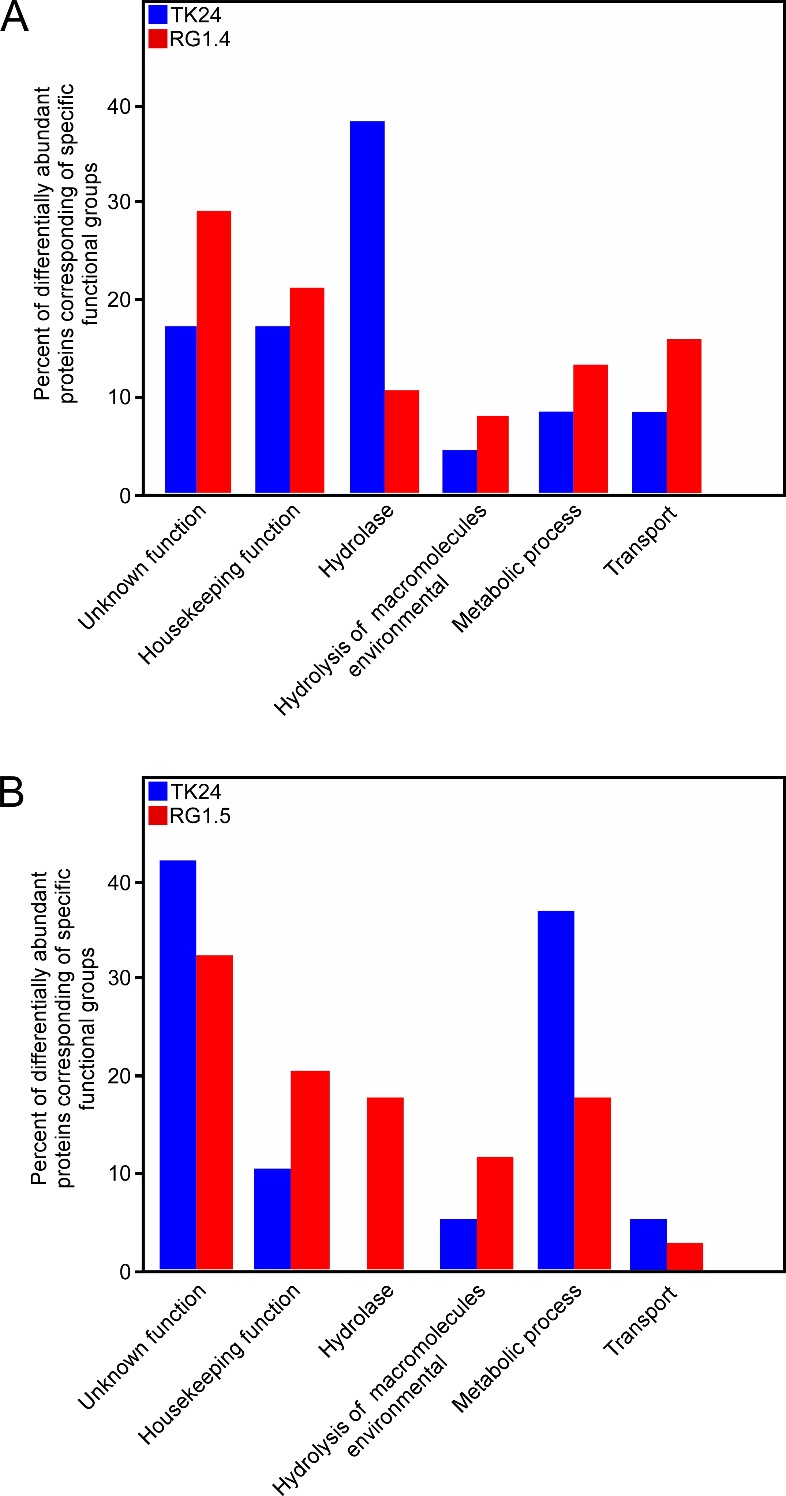


**Figure S4: Functional characterization of the reduced genome strains secretome.**

**(A)** and **(B)** Differentially abundant proteins between TK24 and the reduced genome strains RG1.4 **(A)** and RG1.5 **(B)** are divided into groups based on their function ([Tsolis et al., 2018](#_ENREF_6)). The dataset is filtered to secreted proteins so that cytoplasmic contaminating proteins are removed.


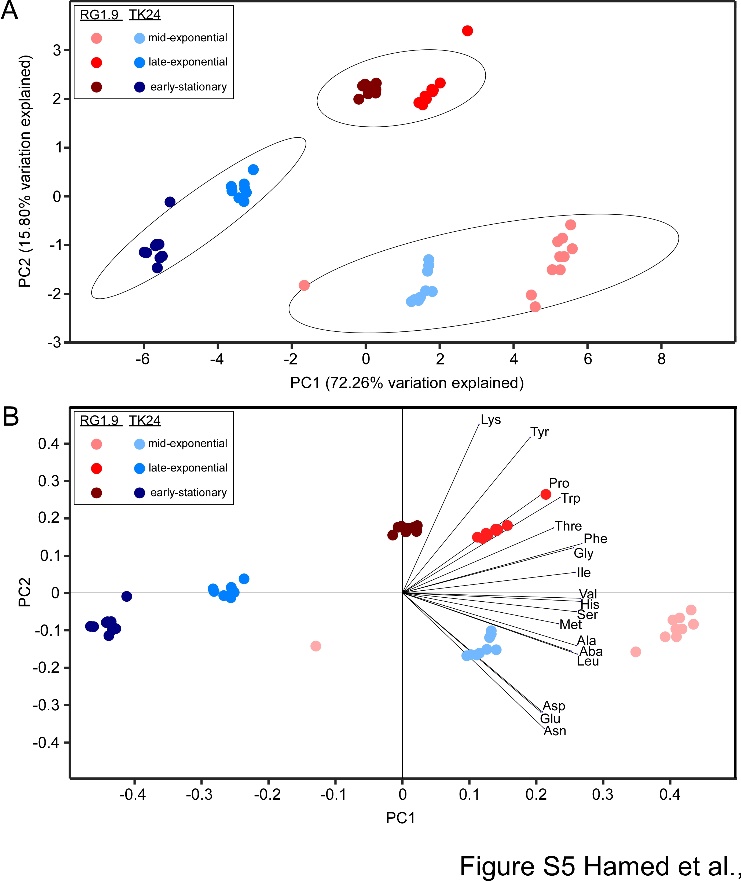


**Figure S5: Exometabolomics changes between strains *S. lividans* TK24 and RG1.9.**

Principle component analysis was performed on amino acid concentrations in the medium measured in different growth phases**. (A)** PCA score plot with 95% confidence ellipses. (**B**) PCA biplot combining the score plot with loadings of the variables. Ala (alanine), Gly (glycine), Aba (α-amino-butyric acid), Val (valine), Leu (leucine), Ile (isoleucine), Thr (threonine), Ser (serine), Pro (proline), Asn (asparagine), Asp (aspartate), Met (methionine), Glu (glutamate), Phe (phenylalanine), Lys (lysing), His (histidine), Tyr (tyrosine), Trp (tryptophan) were used as variables and the experimental values as observations. Data from RG 1.9 and WT are printed in red and blue, respectively. Increasing color intensities corresponds to samples after 8 h (light ; mid-exponential phase), 16hrs (medium; late exponential phase) and 20hrs (dark; early stationary phase).


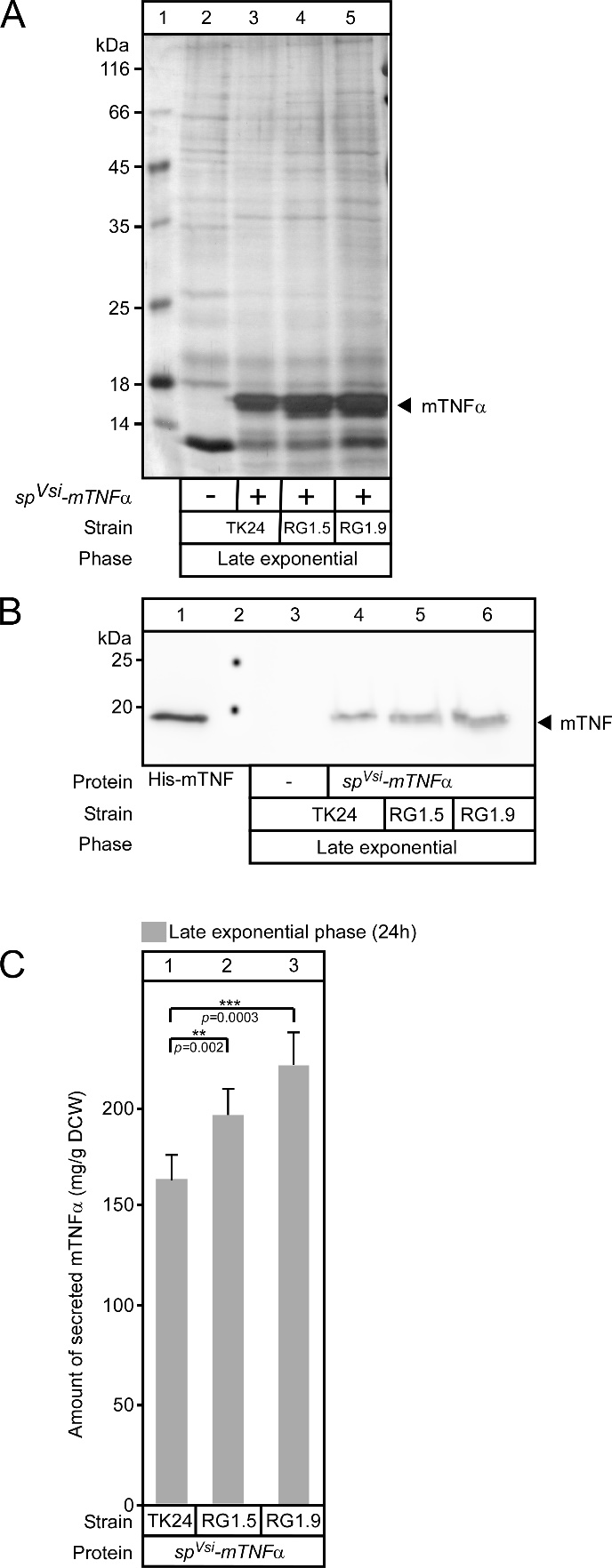


**Figure S6: Effect of deleting specialized metabolites clusters/genes on heterologous protein secretion**

**(A)** Polypeptides from culture supernatants (2-16 µL/lane; equivalent to 0.1 mg DCW) of TK24 or the reduced genome strains carrying a plasmid-borne copy *sp^Secv^-mTNF,* grown in nutrient broth (NB) until late exponential phase were analyzed by SDS-PAGE and silver stained. Lane 1: Molecular weight marker as in (Fig. 2B); secreted mTNF is indicated.

**(B**) Immunostaining of the samples presented in Panel A (same sample loading) using mTNF specific antibodies. Lane 1: purified his-mRFP; Lane 2: molecular weight marker.

**(C)** The amounts of mTNF secreted in nutrient broth (NB) by the indicated strains were quantified following immunodetection (as in A-B). *n*=3, values represent the mean ± SD.

Supplementary tables

**Table S1** Deleted specialized metabolite genes clusters and genes in reduced genome strains

| **Cluster name abbreviation** | **Predicted products of clusters and genes** | **Cluster and genes locations** |
| --- | --- | --- |
| ***Cpk*** | the antibiotic coelimycin P1 and the neighbouring region containing *scbA* and *scbR* genes encoding for production of gammabutyrolactone system | SLIV_06705 to SLIV_06820 |
| ***Red*** | The red-pigmented antibiotic undecylprodigiosin | SLIV_09115 to SLIV_09220 |
| ***Act*** | The blue-pigmented polyketide antibiotic actinorhodin | SLIV_12925 to SLIV_13030 |
| ***Cda*** | The calcium dependent antibiotic | SLIV_21445 to SLIV_21640 |
| ***Mel*** | the brown pigment melanin | SLIV_24135 to SLIV_24180 |
| ***hrdD*** | RNA polymerase sigma factor HrdD | SLIV_21680 |
| ***matAB*** | Extracellular polymeric substance (EPS) (poly β-1,6-N-acetylglucosamine) | SLIV_22850 to SLIV_22890 |

**Table S2: Transcriptomics results for *S. lividans* TK24 selected specialized metabolite gene clusters and genes in MM and MMCAS**.(xlsx)

**Table S3: Proteins identified by MS.**

Proteins identified by mass spectrometric workflow (see Methods). Quantitative values (determined using iBAQ), Sequence coverage for the identified proteins and number of peptides per protein for each biological sample are included.

Table S3 is provided in the additional spreadsheet file:

“Table S3_Proteins identified by mass spectrometry”. (xlsx)

**Table S4: Differentially abundant proteins.**

Comparison of TK24 vs reduced genome strains for differentially abundant proteins, for the secreted proteins based on annotation in the STOPS database <http://www.stopsdb.eu> ([Tsolis et al., 2018](#_ENREF_6)).

Table S4 is provided in the additional spreadsheet file:

“Table S4_Differentially abundant secreted proteins between TK24 and reduced genome strains”.

**Table S5: Statistical significance for identified secreted proteins TK24 and reduced genome strains.**

# Identified secreted proteins in *S. lividans* TK24 and its mutated derivative strains were tested for statistical significance using one-way ANOVA for iBAQ values of each protein followed by a post-hoc test for the significant proteins p-value <0.05. The p-value for post-hoc test is adjusted based on Bonferroni correction.

**Table S6: Transcriptomic analysis for S. lividans TK24 and RG1.9.**

The RNAseq dataset and differential expression of TK24 wild type and RG1.9. The signal intensity value (A-value) was calculated by the average (log_2_) RPKM of each gene and the signal intensity ratio (M-value) by the difference of (log_2_) TPM. The differential RNA-Seq data was evaluated using an adjusted P-value cut-off of P ≤ 0.05 and a signal intensity ratio (M-value) cut-off of ≥ +1 or ≤ − 1 (fold-change of ±2). The genes are grouped based on their functions in different sheets. Genes of the Sig^E^ regulon are marked with one asterisk; genes of the Sig^R^ regulon are marked with two asterisks and genes of the arginine biosynthesis pathway are marked with three asterisks.

**Table S7: Amino acid consumption data for TK24 and RG1.9.**

Supplementary materials and methods

Transcriptomics sample preparation and analysis for TK24 in minimal medium

Samples For transcriptomics data analysis were taken during the mid-log and late-log growth phase as well as after entry into the stationary phase. Harvesting and RNA isolation was performed as described previously ([Busche et al., 2012](#_ENREF_1); [Tsolis et al., 2019](#_ENREF_5)). Samples two different biological replicates, for each experimental condition tested, were isolated separately and pooled after quality control. The RNA quality was checked via Agilent 2100 Bioanalyzer (Agilent Technologies, Böblingen, Germany) and Trinean Xposesystem (Gent, Belgium) prior and after rRNA depletion using the Ribo Zero rRNA Removal Kit for Bacteria (Epicentre, Madison, WI). The TruSeq Stranded mRNA Library Prep Kit from Illumina was used to prepare the cDNA libraries, which were then sequenced in paired-end mode on an Illumina HiSeq 1500 system with 28 respectively 70 bases read length.

Transcripts per kilobase million (TPM) ([Wagner et al., 2012](#_ENREF_7)) were calculated using ReadXplorer v.2.2 ([Hilker et al., 2016](#_ENREF_4)). For differential RNA-Seq analyses the signal intensity value (A-value) was calculated by average log_2_ (TPM) of each gene and the signal intensity ratio (M-value) by the difference of log_2_ (TPM). In cases where the TPM for a gene was 0, a TPM of 0.1 was used instead to avoid log_2_ (0). To identify proteases that were strongly transcribed and differentially expressed under at least one condition, the RNA-Seq data were filtered using a TPM cut-off of 100 and an M-value cut-off of >1.0 under at least one condition.

Supplementary Results

A reduced genome strain displays metabolic alterations

To understand the difference in recombinant protein expression capacity between TK24 and RG1.9, we looked for differences in the exometabolomic profiles of both strains in NB medium, rich with amino acids which serve as carbon and nitrogen source for growth and protein formation. Free amino acid concentrations were analyzed for each strain in the mid-exponential, late-exponential and early stationary phases. To analyze the patterns in these time series data, a principle component analysis was performed. Two principle components explain 88% of the variance in the data (Fig. 5A). The metabolomic pattern diverges over time. Mid exponential data still cluster together for both strains, whereas later time points cluster together depending on the strain. Loadings of the amino acids (Fig. 5B) demonstrate how each variable (amino acid) influences the principal components. Aspartate, glutamate and asparagine strongly correlate and explain the clustering of the metabolomic footprint of both strains in the exponential growth phase. All three amino acids are rapidly consumed, and aspartate and glutamate are depleted after the mid-exponential phase (Table S7), as seen previously ([D'Huys et al., 2011](#_ENREF_2); [D'Huys et al., 2012](#_ENREF_3)).

Lysine, proline, tyrosine and tryptophan are poorly correlated to the other amino acids, and concentration data show no uptake of these amino acids. Isoleucine, methionine, valine, histidine explain part of PC1 and threonine, phenylalanine and glycine can also be grouped and contribute to PC1 and PC2. Late exponential phase and early stationary phase seem to distinguish mostly for these two groups of amino acids. Although it is not possible to accurately estimate specific uptake rates, differences in uptake rates seem to exist between TK24 and RG1.9. The changed secondary metabolism in RG1.9 is most likely the explanation for the altered uptake patterns but it is not possible to retrieve exact correlations with metabolic pathways of secondary metabolism.

**References:**

Busche, T., Silar, R., Picmanova, M., Patek, M., and Kalinowski, J. (2012). Transcriptional regulation of the operon encoding stress-responsive ECF sigma factor SigH and its anti-sigma factor RshA, and control of its regulatory network in Corynebacterium glutamicum. *BMC Genomics* 13**,** 445. doi: 10.1186/1471-2164-13-445.

D'Huys, P.J., Lule, I., Van Hove, S., Vercammen, D., Wouters, C., Bernaerts, K., et al. (2011). Amino acid uptake profiling of wild type and recombinant Streptomyces lividans TK24 batch fermentations. *J Biotechnol* 152(4)**,** 132-143. doi: 10.1016/j.jbiotec.2010.08.011.

D'Huys, P.J., Lule, I., Vercammen, D., Anné, J., Van Impe, J.F., and Bernaerts, K. (2012). Genome-scale metabolic flux analysis of Streptomyces lividans growing on a complex medium. *J Biotechnol* 161(1)**,** 1-13. doi: 10.1016/j.jbiotec.2012.04.010.

Hilker, R., Stadermann, K.B., Schwengers, O., Anisiforov, E., Jaenicke, S., Weisshaar, B., et al. (2016). ReadXplorer 2-detailed read mapping analysis and visualization from one single source. *Bioinformatics* 32(24)**,** 3702-3708. doi: 10.1093/bioinformatics/btw541.

Tsolis, K.C., Hamed, M.B., Simoens, K., Koepff, J., Busche, T., Ruckert, C., et al. (2019). Secretome Dynamics in a Gram-Positive Bacterial Model. 18(3)**,** 423-436. doi: 10.1074/mcp.RA118.000899.

Tsolis, K.C., Tsare, E.P., Orfanoudaki, G., Busche, T., Kanaki, K., Ramakrishnan, R., et al. (2018). Comprehensive subcellular topologies of polypeptides in *Streptomyces*. *Microb Cell Fact* 17(1)**,** 43. doi: 10.1186/s12934-018-0892-0.

Wagner, G.P., Kin, K., and Lynch, V.J. (2012). Measurement of mRNA abundance using RNA-seq data: RPKM measure is inconsistent among samples. *Theory Biosci* 131(4)**,** 281-285. doi: 10.1007/s12064-012-0162-3.
